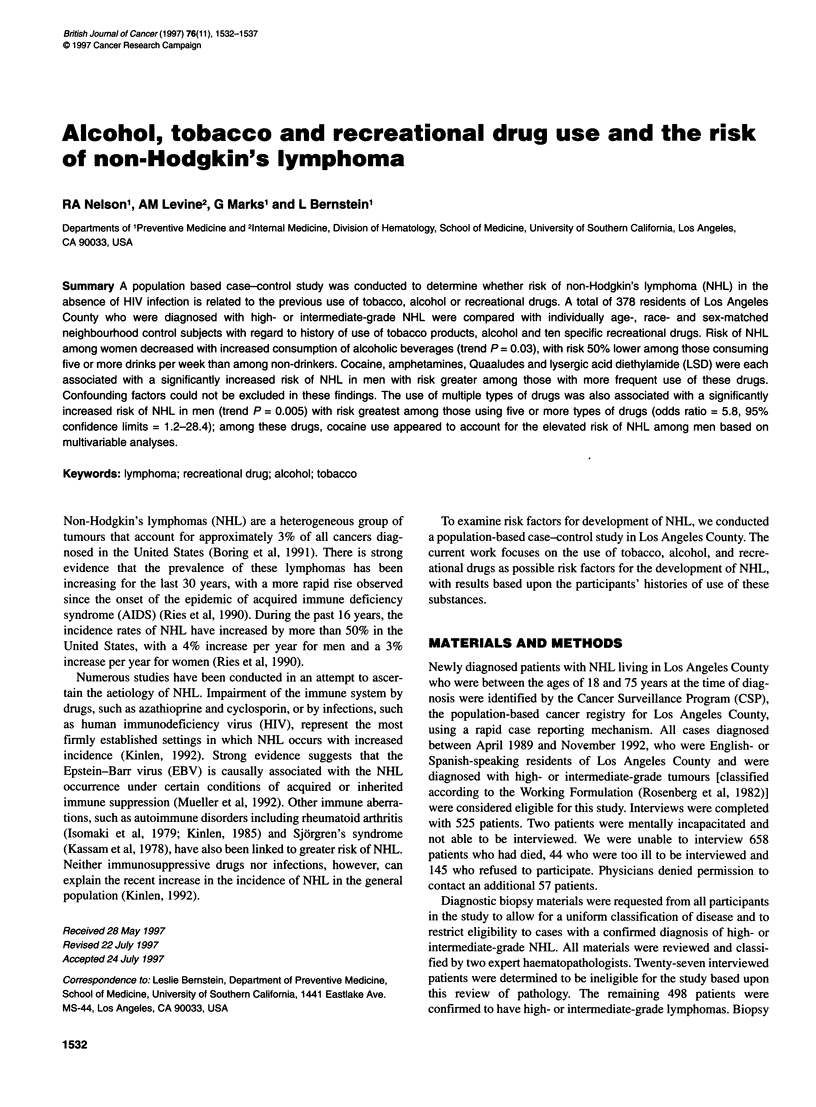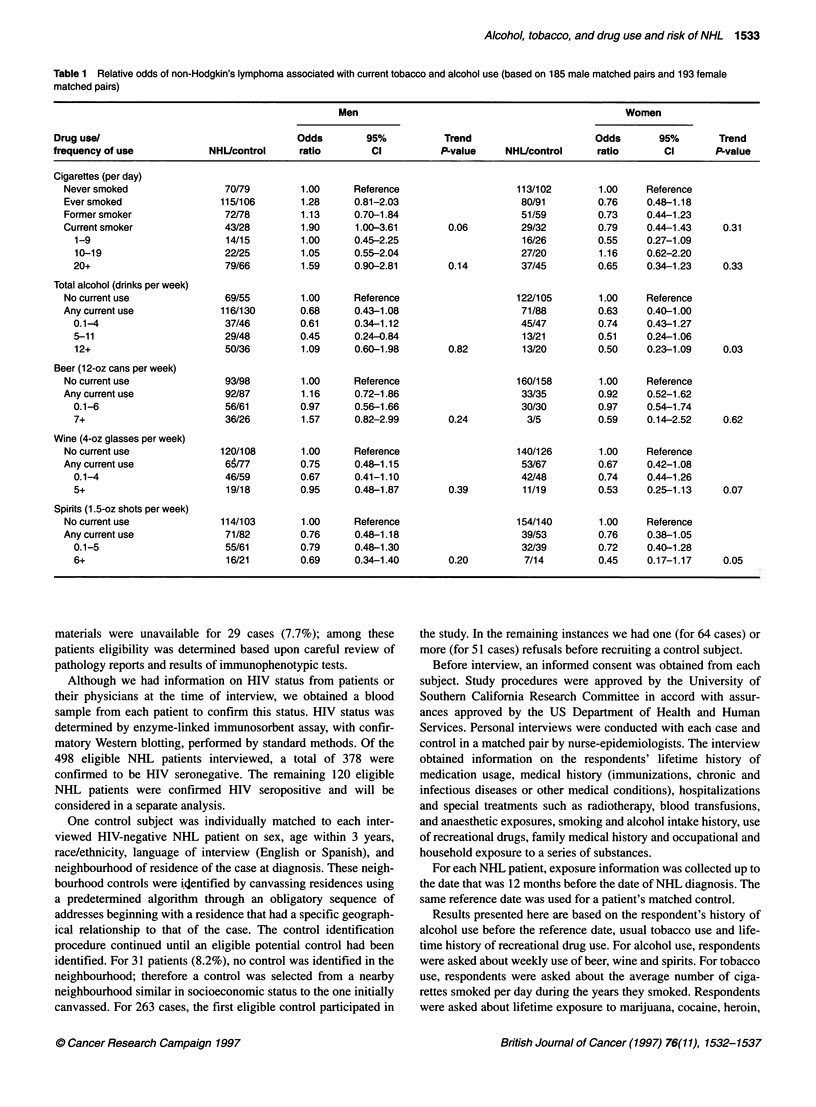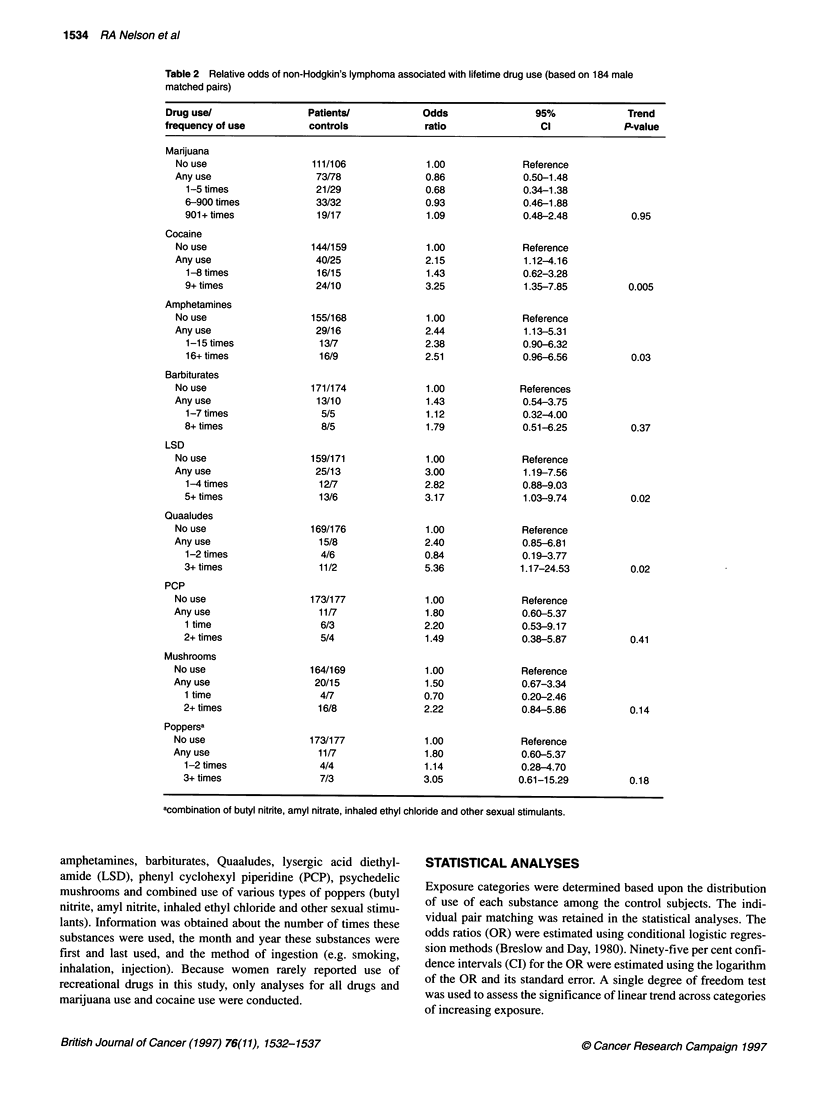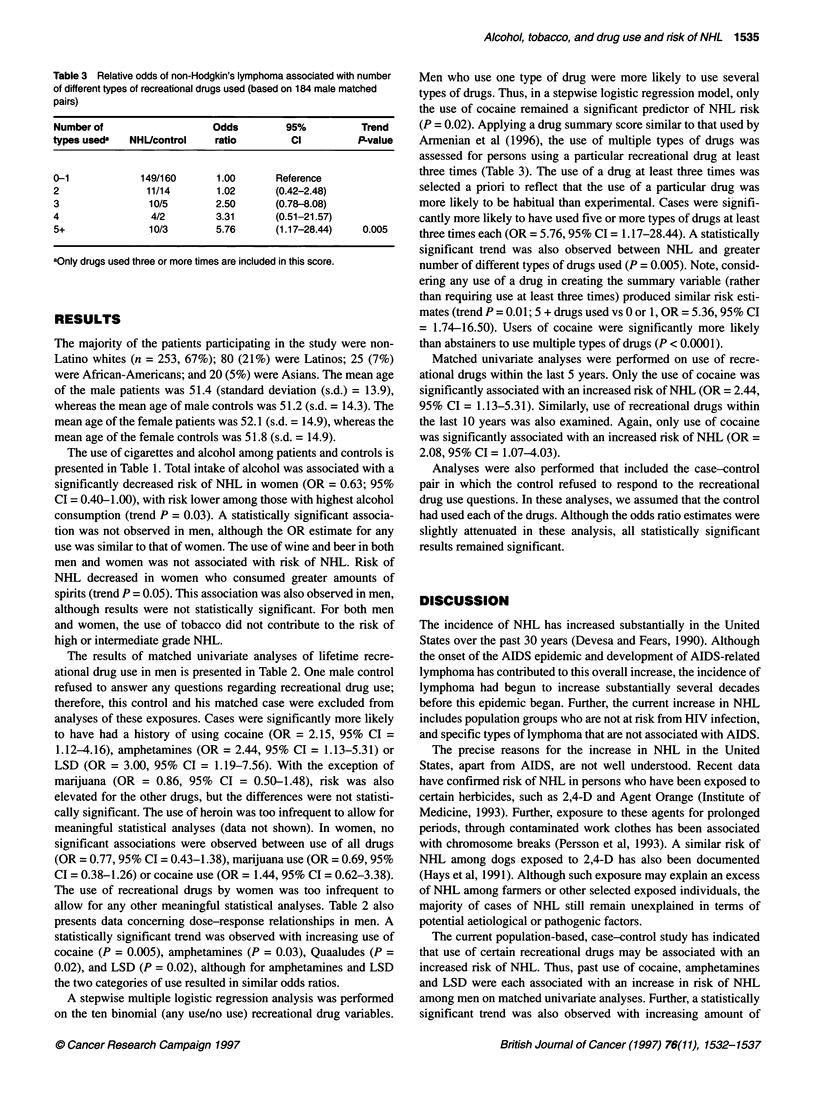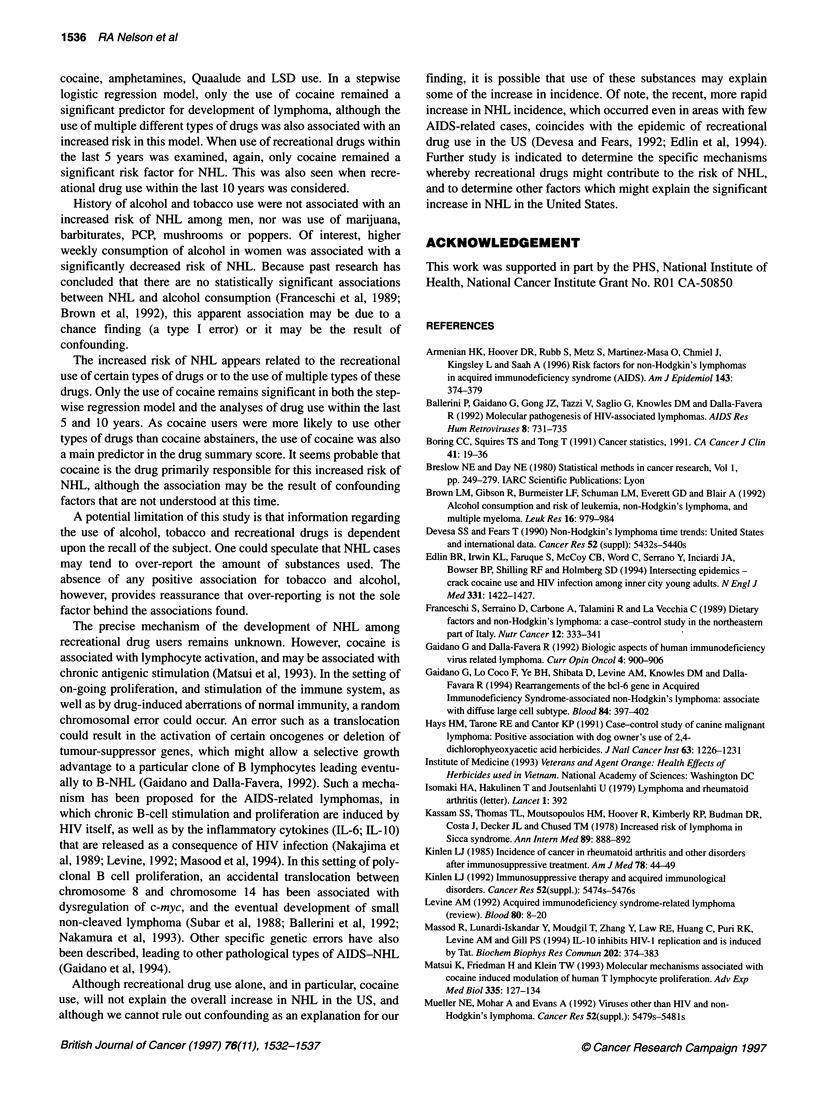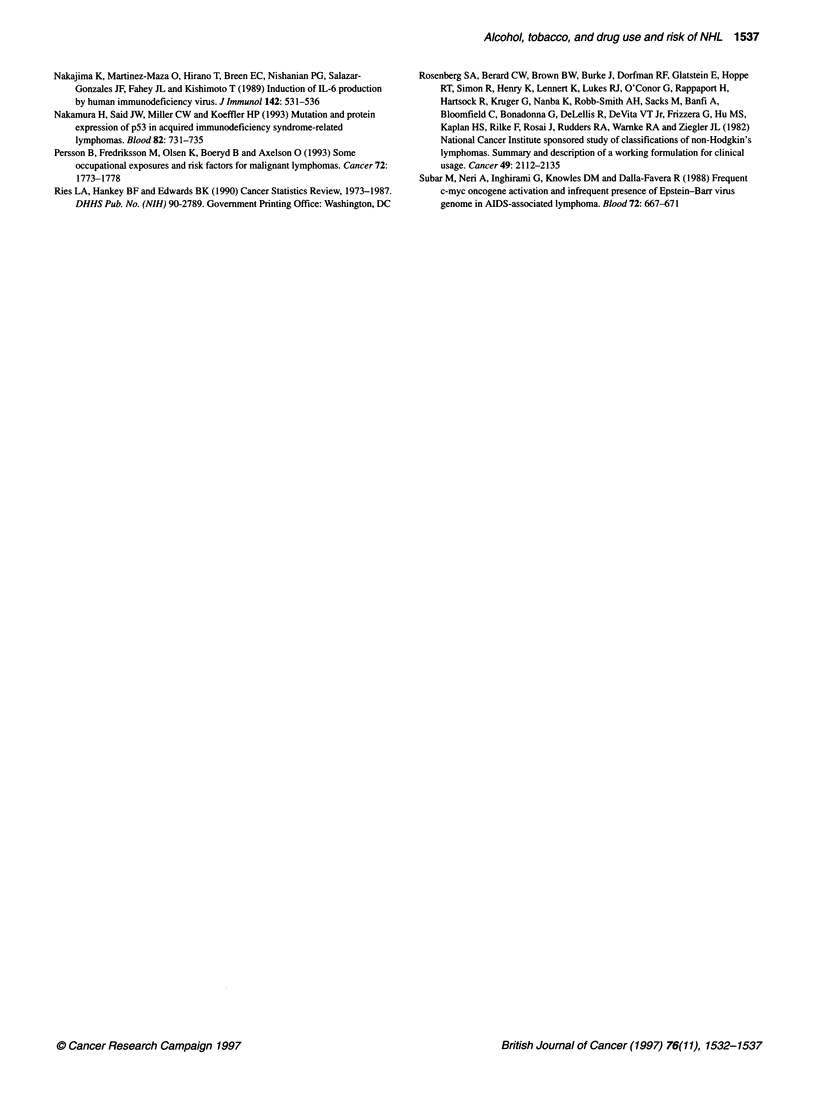# Alcohol, tobacco and recreational drug use and the risk of non-Hodgkin's lymphoma.

**DOI:** 10.1038/bjc.1997.590

**Published:** 1997

**Authors:** R. A. Nelson, A. M. Levine, G. Marks, L. Bernstein

**Affiliations:** Department of Preventive Medicine, School of Medicine, University of Southern California, Los Angeles 90033, USA.

## Abstract

A population based case-control study was conducted to determine whether risk of non-Hodgkin's lymphoma (NHL) in the absence of HIV infection is related to the previous use of tobacco, alcohol or recreational drugs. A total of 378 residents of Los Angeles County who were diagnosed with high- or intermediate-grade NHL were compared with individually age-, race- and sex-matched neighbourhood control subjects with regard to history of use of tobacco products, alcohol and ten specific recreational drugs. Risk of NHL among women decreased with increased consumption of alcoholic beverages (trend P = 0.03), with risk 50% lower among those consuming five or more drinks per week than among non-drinkers. Cocaine, amphetamines, Quaaludes and lysergic acid diethylamide (LSD) were each associated with a significantly increased risk of NHL in men with risk greater among those with more frequent use of these drugs. Confounding factors could not be excluded in these findings. The use of multiple types of drugs was also associated with a significantly increased risk of NHL in men (trend P = 0.005) with risk greatest among those using five or more types of drugs (odds ratio = 5.8, 95% confidence limits = 1.2-28.4); among these drugs, cocaine use appeared to account for the elevated risk of NHL among men based on multivariable analyses.


British Journal of Cancer (1997) 76(11), 1532–1537
© 1997 Cancer Research Campaign

### Alcohol, tobacco and recreational drug use and the risk of non-Hodgkin's lymphoma

**RA Nelson[1], AM Levine[2], G Marks[1] and L Bernstein[1]**

Departments of [1]Preventive Medicine and [2]Internal Medicine, Division of Hematology, School of Medicine, University of Southern California, Los Angeles, CA 90033, USA

**Summary** A population based case–control study was conducted to determine whether risk of non-Hodgkin's lymphoma (NHL) in the absence of HIV infection is related to the previous use of tobacco, alcohol or recreational drugs. A total of 378 residents of Los Angeles County who were diagnosed with high- or intermediate-grade NHL were compared with individually age-, race- and sex-matched neighbourhood control subjects with regard to history of use of tobacco products, alcohol and ten specific recreational drugs. Risk of NHL among women decreased with increased consumption of alcoholic beverages (trend $P = 0.03$), with risk 50% lower among those consuming five or more drinks per week than among non-drinkers. Cocaine, amphetamines, Quaaludes and lysergic acid diethylamide (LSD) were each associated with a significantly increased risk of NHL in men with risk greater among those with more frequent use of these drugs. Confounding factors could not be excluded in these findings. The use of multiple types of drugs was also associated with a significantly increased risk of NHL in men (trend $P = 0.005$) with risk greatest among those using five or more types of drugs (odds ratio = 5.8, 95% confidence limits = 1.2–28.4); among these drugs, cocaine use appeared to account for the elevated risk of NHL among men based on multivariable analyses.

**Keywords:** lymphoma; recreational drug; alcohol; tobacco

Non-Hodgkin's lymphomas (NHL) are a heterogeneous group of tumours that account for approximately 3% of all cancers diagnosed in the United States (Boring et al, 1991). There is strong evidence that the prevalence of these lymphomas has been increasing for the last 30 years, with a more rapid rise observed since the onset of the epidemic of acquired immune deficiency syndrome (AIDS) (Ries et al, 1990). During the past 16 years, the incidence rates of NHL have increased by more than 50% in the United States, with a 4% increase per year for men and a 3% increase per year for women (Ries et al, 1990).

Numerous studies have been conducted in an attempt to ascertain the aetiology of NHL. Impairment of the immune system by drugs, such as azathioprine and cyclosporin, or by infections, such as human immunodeficiency virus (HIV), represent the most firmly established settings in which NHL occurs with increased incidence (Kinlen, 1992). Strong evidence suggests that the Epstein–Barr virus (EBV) is causally associated with the NHL occurrence under certain conditions of acquired or inherited immune suppression (Mueller et al, 1992). Other immune aberrations, such as autoimmune disorders including rheumatoid arthritis (Isomaki et al, 1979; Kinlen, 1985) and Sjörgren's syndrome (Kassam et al, 1978), have also been linked to greater risk of NHL. Neither immunosuppressive drugs nor infections, however, can explain the recent increase in the incidence of NHL in the general population (Kinlen, 1992).

*Received 28 May 1997*
*Revised 22 July 1997*
*Accepted 24 July 1997*

*Correspondence to:* Leslie Bernstein, Department of Preventive Medicine, School of Medicine, University of Southern California, 1441 Eastlake Ave. MS-44, Los Angeles, CA 90033, USA

To examine risk factors for development of NHL, we conducted a population-based case–control study in Los Angeles County. The current work focuses on the use of tobacco, alcohol, and recreational drugs as possible risk factors for the development of NHL, with results based upon the participants' histories of use of these substances.

### MATERIALS AND METHODS

Newly diagnosed patients with NHL living in Los Angeles County who were between the ages of 18 and 75 years at the time of diagnosis were identified by the Cancer Surveillance Program (CSP), the population-based cancer registry for Los Angeles County, using a rapid case reporting mechanism. All cases diagnosed between April 1989 and November 1992, who were English- or Spanish-speaking residents of Los Angeles County and were diagnosed with high- or intermediate-grade tumours [classified according to the Working Formulation (Rosenberg et al, 1982)] were considered eligible for this study. Interviews were completed with 525 patients. Two patients were mentally incapacitated and not able to be interviewed. We were unable to interview 658 patients who had died, 44 who were too ill to be interviewed and 145 who refused to participate. Physicians denied permission to contact an additional 57 patients.

Diagnostic biopsy materials were requested from all participants in the study to allow for a uniform classification of disease and to restrict eligibility to cases with a confirmed diagnosis of high- or intermediate-grade NHL. All materials were reviewed and classified by two expert haematopathologists. Twenty-seven interviewed patients were determined to be ineligible for the study based upon this review of pathology. The remaining 498 patients were confirmed to have high- or intermediate-grade lymphomas. Biopsy

**Table 1** Relative odds of non-Hodgkin's lymphoma associated with current tobacco and alcohol use (based on 185 male matched pairs and 193 female matched pairs)

| Drug use/ frequency of use | Men | | | | Women | | | |
|---|---|---|---|---|---|---|---|---|
| | NHL/control | Odds ratio | 95% CI | Trend *P*-value | NHL/control | Odds ratio | 95% CI | Trend *P*-value |
| Cigarettes (per day) | | | | | | | | |
| Never smoked | 70/79 | 1.00 | Reference | | 113/102 | 1.00 | Reference | |
| Ever smoked | 115/106 | 1.28 | 0.81–2.03 | | 80/91 | 0.76 | 0.48–1.18 | |
| Former smoker | 72/78 | 1.13 | 0.70–1.84 | | 51/59 | 0.73 | 0.44–1.23 | |
| Current smoker | 43/28 | 1.90 | 1.00–3.61 | 0.06 | 29/32 | 0.79 | 0.44–1.43 | 0.31 |
| 1–9 | 14/15 | 1.00 | 0.45–2.25 | | 16/26 | 0.55 | 0.27–1.09 | |
| 10–19 | 22/25 | 1.05 | 0.55–2.04 | | 27/20 | 1.16 | 0.62–2.20 | |
| 20+ | 79/66 | 1.59 | 0.90–2.81 | 0.14 | 37/45 | 0.65 | 0.34–1.23 | 0.33 |
| Total alcohol (drinks per week) | | | | | | | | |
| No current use | 69/55 | 1.00 | Reference | | 122/105 | 1.00 | Reference | |
| Any current use | 116/130 | 0.68 | 0.43–1.08 | | 71/88 | 0.63 | 0.40–1.00 | |
| 0.1–4 | 37/46 | 0.61 | 0.34–1.12 | | 45/47 | 0.74 | 0.43–1.27 | |
| 5–11 | 29/48 | 0.45 | 0.24–0.84 | | 13/21 | 0.51 | 0.24–1.06 | |
| 12+ | 50/36 | 1.09 | 0.60–1.98 | 0.82 | 13/20 | 0.50 | 0.23–1.09 | 0.03 |
| Beer (12-oz cans per week) | | | | | | | | |
| No current use | 93/98 | 1.00 | Reference | | 160/158 | 1.00 | Reference | |
| Any current use | 92/87 | 1.16 | 0.72–1.86 | | 33/35 | 0.92 | 0.52–1.62 | |
| 0.1–6 | 56/61 | 0.97 | 0.56–1.66 | | 30/30 | 0.97 | 0.54–1.74 | |
| 7+ | 36/26 | 1.57 | 0.82–2.99 | 0.24 | 3/5 | 0.59 | 0.14–2.52 | 0.62 |
| Wine (4-oz glasses per week) | | | | | | | | |
| No current use | 120/108 | 1.00 | Reference | | 140/126 | 1.00 | Reference | |
| Any current use | 65/77 | 0.75 | 0.48–1.15 | | 53/67 | 0.67 | 0.42–1.08 | |
| 0.1–4 | 46/59 | 0.67 | 0.41–1.10 | | 42/48 | 0.74 | 0.44–1.26 | |
| 5+ | 19/18 | 0.95 | 0.48–1.87 | 0.39 | 11/19 | 0.53 | 0.25–1.13 | 0.07 |
| Spirits (1.5-oz shots per week) | | | | | | | | |
| No current use | 114/103 | 1.00 | Reference | | 154/140 | 1.00 | Reference | |
| Any current use | 71/82 | 0.76 | 0.48–1.18 | | 39/53 | 0.76 | 0.38–1.05 | |
| 0.1–5 | 55/61 | 0.79 | 0.48–1.30 | | 32/39 | 0.72 | 0.40–1.28 | |
| 6+ | 16/21 | 0.69 | 0.34–1.40 | 0.20 | 7/14 | 0.45 | 0.17–1.17 | 0.05 |

materials were unavailable for 29 cases (7.7%); among these patients eligibility was determined based upon careful review of pathology reports and results of immunophenotypic tests.

Although we had information on HIV status from patients or their physicians at the time of interview, we obtained a blood sample from each patient to confirm this status. HIV status was determined by enzyme-linked immunosorbent assay, with confirmatory Western blotting, performed by standard methods. Of the 498 eligible NHL patients interviewed, a total of 378 were confirmed to be HIV seronegative. The remaining 120 eligible NHL patients were confirmed HIV seropositive and will be considered in a separate analysis.

One control subject was individually matched to each interviewed HIV-negative NHL patient on sex, age within 3 years, race/ethnicity, language of interview (English or Spanish), and neighbourhood of residence of the case at diagnosis. These neighbourhood controls were identified by canvassing residences using a predetermined algorithm through an obligatory sequence of addresses beginning with a residence that had a specific geographical relationship to that of the case. The control identification procedure continued until an eligible potential control had been identified. For 31 patients (8.2%), no control was identified in the neighbourhood; therefore a control was selected from a nearby neighbourhood similar in socioeconomic status to the one initially canvassed. For 263 cases, the first eligible control participated in the study. In the remaining instances we had one (for 64 cases) or more (for 51 cases) refusals before recruiting a control subject.

Before interview, an informed consent was obtained from each subject. Study procedures were approved by the University of Southern California Research Committee in accord with assurances approved by the US Department of Health and Human Services. Personal interviews were conducted with each case and control in a matched pair by nurse-epidemiologists. The interview obtained information on the respondents' lifetime history of medication usage, medical history (immunizations, chronic and infectious diseases or other medical conditions), hospitalizations and special treatments such as radiotherapy, blood transfusions, and anaesthetic exposures, smoking and alcohol intake history, use of recreational drugs, family medical history and occupational and household exposure to a series of substances.

For each NHL patient, exposure information was collected up to the date that was 12 months before the date of NHL diagnosis. The same reference date was used for a patient's matched control.

Results presented here are based on the respondent's history of alcohol use before the reference date, usual tobacco use and lifetime history of recreational drug use. For alcohol use, respondents were asked about weekly use of beer, wine and spirits. For tobacco use, respondents were asked about the average number of cigarettes smoked per day during the years they smoked. Respondents were asked about lifetime exposure to marijuana, cocaine, heroin,

© Cancer Research Campaign 1997

**Table 2** Relative odds of non-Hodgkin's lymphoma associated with lifetime drug use (based on 184 male matched pairs)

| Drug use/ frequency of use | Patients/ controls | Odds ratio | 95% CI | Trend P-value |
|---|---|---|---|---|
| Marijuana | | | | |
| No use | 111/106 | 1.00 | Reference | |
| Any use | 73/78 | 0.86 | 0.50–1.48 | |
| 1–5 times | 21/29 | 0.68 | 0.34–1.38 | |
| 6–900 times | 33/32 | 0.93 | 0.46–1.88 | |
| 901+ times | 19/17 | 1.09 | 0.48–2.48 | 0.95 |
| Cocaine | | | | |
| No use | 144/159 | 1.00 | Reference | |
| Any use | 40/25 | 2.15 | 1.12–4.16 | |
| 1–8 times | 16/15 | 1.43 | 0.62–3.28 | |
| 9+ times | 24/10 | 3.25 | 1.35–7.85 | 0.005 |
| Amphetamines | | | | |
| No use | 155/168 | 1.00 | Reference | |
| Any use | 29/16 | 2.44 | 1.13–5.31 | |
| 1–15 times | 13/7 | 2.38 | 0.90–6.32 | |
| 16+ times | 16/9 | 2.51 | 0.96–6.56 | 0.03 |
| Barbiturates | | | | |
| No use | 171/174 | 1.00 | References | |
| Any use | 13/10 | 1.43 | 0.54–3.75 | |
| 1–7 times | 5/5 | 1.12 | 0.32–4.00 | |
| 8+ times | 8/5 | 1.79 | 0.51–6.25 | 0.37 |
| LSD | | | | |
| No use | 159/171 | 1.00 | Reference | |
| Any use | 25/13 | 3.00 | 1.19–7.56 | |
| 1–4 times | 12/7 | 2.82 | 0.88–9.03 | |
| 5+ times | 13/6 | 3.17 | 1.03–9.74 | 0.02 |
| Quaaludes | | | | |
| No use | 169/176 | 1.00 | Reference | |
| Any use | 15/8 | 2.40 | 0.85–6.81 | |
| 1–2 times | 4/6 | 0.84 | 0.19–3.77 | |
| 3+ times | 11/2 | 5.36 | 1.17–24.53 | 0.02 |
| PCP | | | | |
| No use | 173/177 | 1.00 | Reference | |
| Any use | 11/7 | 1.80 | 0.60–5.37 | |
| 1 time | 6/3 | 2.20 | 0.53–9.17 | |
| 2+ times | 5/4 | 1.49 | 0.38–5.87 | 0.41 |
| Mushrooms | | | | |
| No use | 164/169 | 1.00 | Reference | |
| Any use | 20/15 | 1.50 | 0.67–3.34 | |
| 1 time | 4/7 | 0.70 | 0.20–2.46 | |
| 2+ times | 16/8 | 2.22 | 0.84–5.86 | 0.14 |
| Poppers[a] | | | | |
| No use | 173/177 | 1.00 | Reference | |
| Any use | 11/7 | 1.80 | 0.60–5.37 | |
| 1–2 times | 4/4 | 1.14 | 0.28–4.70 | |
| 3+ times | 7/3 | 3.05 | 0.61–15.29 | 0.18 |

[a]combination of butyl nitrite, amyl nitrate, inhaled ethyl chloride and other sexual stimulants.

amphetamines, barbiturates, Quaaludes, lysergic acid diethylamide (LSD), phenyl cyclohexyl piperidine (PCP), psychedelic mushrooms and combined use of various types of poppers (butyl nitrite, amyl nitrite, inhaled ethyl chloride and other sexual stimulants). Information was obtained about the number of times these substances were used, the month and year these substances were first and last used, and the method of ingestion (e.g. smoking, inhalation, injection). Because women rarely reported use of recreational drugs in this study, only analyses for all drugs and marijuana use and cocaine use were conducted.

### STATISTICAL ANALYSES

Exposure categories were determined based upon the distribution of use of each substance among the control subjects. The individual pair matching was retained in the statistical analyses. The odds ratios (OR) were estimated using conditional logistic regression methods (Breslow and Day, 1980). Ninety-five per cent confidence intervals (CI) for the OR were estimated using the logarithm of the OR and its standard error. A single degree of freedom test was used to assess the significance of linear trend across categories of increasing exposure.

© Cancer Research Campaign 1997

**Table 3** Relative odds of non-Hodgkin's lymphoma associated with number of different types of recreational drugs used (based on 184 male matched pairs)

| Number of types used[a] | NHL/control | Odds ratio | 95% CI | Trend *P*-value |
|---|---|---|---|---|
| 0–1 | 149/160 | 1.00 | Reference | |
| 2 | 11/14 | 1.02 | (0.42–2.48) | |
| 3 | 10/5 | 2.50 | (0.78–8.08) | |
| 4 | 4/2 | 3.31 | (0.51–21.57) | |
| 5+ | 10/3 | 5.76 | (1.17–28.44) | 0.005 |

[a]Only drugs used three or more times are included in this score.

### RESULTS

The majority of the patients participating in the study were non-Latino whites ($n$ = 253, 67%); 80 (21%) were Latinos; 25 (7%) were African-Americans; and 20 (5%) were Asians. The mean age of the male patients was 51.4 (standard deviation (s.d.) = 13.9), whereas the mean age of male controls was 51.2 (s.d. = 14.3). The mean age of the female patients was 52.1 (s.d. = 14.9), whereas the mean age of the female controls was 51.8 (s.d. = 14.9).

The use of cigarettes and alcohol among patients and controls is presented in Table 1. Total intake of alcohol was associated with a significantly decreased risk of NHL in women (OR = 0.63; 95% CI = 0.40–1.00), with risk lower among those with highest alcohol consumption (trend $P$ = 0.03). A statistically significant association was not observed in men, although the OR estimate for any use was similar to that of women. The use of wine and beer in both men and women was not associated with risk of NHL. Risk of NHL decreased in women who consumed greater amounts of spirits (trend $P$ = 0.05). This association was also observed in men, although results were not statistically significant. For both men and women, the use of tobacco did not contribute to the risk of high or intermediate grade NHL.

The results of matched univariate analyses of lifetime recreational drug use in men is presented in Table 2. One male control refused to answer any questions regarding recreational drug use; therefore, this control and his matched case were excluded from analyses of these exposures. Cases were significantly more likely to have had a history of using cocaine (OR = 2.15, 95% CI = 1.12–4.16), amphetamines (OR = 2.44, 95% CI = 1.13–5.31) or LSD (OR = 3.00, 95% CI = 1.19–7.56). With the exception of marijuana (OR = 0.86, 95% CI = 0.50–1.48), risk was also elevated for the other drugs, but the differences were not statistically significant. The use of heroin was too infrequent to allow for meaningful statistical analyses (data not shown). In women, no significant associations were observed between use of all drugs (OR = 0.77, 95% CI = 0.43–1.38), marijuana use (OR = 0.69, 95% CI = 0.38–1.26) or cocaine use (OR = 1.44, 95% CI = 0.62–3.38). The use of recreational drugs by women was too infrequent to allow for any other meaningful statistical analyses. Table 2 also presents data concerning dose–response relationships in men. A statistically significant trend was observed with increasing use of cocaine ($P$ = 0.005), amphetamines ($P$ = 0.03), Quaaludes ($P$ = 0.02), and LSD ($P$ = 0.02), although for amphetamines and LSD the two categories of use resulted in similar odds ratios.

A stepwise multiple logistic regression analysis was performed on the ten binomial (any use/no use) recreational drug variables. Men who use one type of drug were more likely to use several types of drugs. Thus, in a stepwise logistic regression model, only the use of cocaine remained a significant predictor of NHL risk ($P$ = 0.02). Applying a drug summary score similar to that used by Armenian et al (1996), the use of multiple types of drugs was assessed for persons using a particular recreational drug at least three times (Table 3). The use of a drug at least three times was selected a priori to reflect that the use of a particular drug was more likely to be habitual than experimental. Cases were significantly more likely to have used five or more types of drugs at least three times each (OR = 5.76, 95% CI = 1.17–28.44). A statistically significant trend was also observed between NHL and greater number of different types of drugs used ($P$ = 0.005). Note, considering any use of a drug in creating the summary variable (rather than requiring use at least three times) produced similar risk estimates (trend $P$ = 0.01; 5 + drugs used vs 0 or 1, OR = 5.36, 95% CI = 1.74–16.50). Users of cocaine were significantly more likely than abstainers to use multiple types of drugs ($P$ < 0.0001).

Matched univariate analyses were performed on use of recreational drugs within the last 5 years. Only the use of cocaine was significantly associated with an increased risk of NHL (OR = 2.44, 95% CI = 1.13–5.31). Similarly, use of recreational drugs within the last 10 years was also examined. Again, only use of cocaine was significantly associated with an increased risk of NHL (OR = 2.08, 95% CI = 1.07–4.03).

Analyses were also performed that included the case–control pair in which the control refused to respond to the recreational drug use questions. In these analyses, we assumed that the control had used each of the drugs. Although the odds ratio estimates were slightly attenuated in these analysis, all statistically significant results remained significant.

### DISCUSSION

The incidence of NHL has increased substantially in the United States over the past 30 years (Devesa and Fears, 1990). Although the onset of the AIDS epidemic and development of AIDS-related lymphoma has contributed to this overall increase, the incidence of lymphoma had begun to increase substantially several decades before this epidemic began. Further, the current increase in NHL includes population groups who are not at risk from HIV infection, and specific types of lymphoma that are not associated with AIDS.

The precise reasons for the increase in NHL in the United States, apart from AIDS, are not well understood. Recent data have confirmed risk of NHL in persons who have been exposed to certain herbicides, such as 2,4-D and Agent Orange (Institute of Medicine, 1993). Further, exposure to these agents for prolonged periods, through contaminated work clothes has been associated with chromosome breaks (Persson et al, 1993). A similar risk of NHL among dogs exposed to 2,4-D has also been documented (Hays et al, 1991). Although such exposure may explain an excess of NHL among farmers or other selected exposed individuals, the majority of cases of NHL still remain unexplained in terms of potential aetiological or pathogenic factors.

The current population-based, case–control study has indicated that use of certain recreational drugs may be associated with an increased risk of NHL. Thus, past use of cocaine, amphetamines and LSD were each associated with an increase in risk of NHL among men on matched univariate analyses. Further, a statistically significant trend was also observed with increasing amount of

© Cancer Research Campaign 1997

cocaine, amphetamines, Quaalude and LSD use. In a stepwise logistic regression model, only the use of cocaine remained a significant predictor for development of lymphoma, although the use of multiple different types of drugs was also associated with an increased risk in this model. When use of recreational drugs within the last 5 years was examined, again, only cocaine remained a significant risk factor for NHL. This was also seen when recreational drug use within the last 10 years was considered.

History of alcohol and tobacco use were not associated with an increased risk of NHL among men, nor was use of marijuana, barbiturates, PCP, mushrooms or poppers. Of interest, higher weekly consumption of alcohol in women was associated with a significantly decreased risk of NHL. Because past research has concluded that there are no statistically significant associations between NHL and alcohol consumption (Franceschi et al, 1989; Brown et al, 1992), this apparent association may be due to a chance finding (a type I error) or it may be the result of confounding.

The increased risk of NHL appears related to the recreational use of certain types of drugs or to the use of multiple types of these drugs. Only the use of cocaine remains significant in both the stepwise regression model and the analyses of drug use within the last 5 and 10 years. As cocaine users were more likely to use other types of drugs than cocaine abstainers, the use of cocaine was also a main predictor in the drug summary score. It seems probable that cocaine is the drug primarily responsible for this increased risk of NHL, although the association may be the result of confounding factors that are not understood at this time.

A potential limitation of this study is that information regarding the use of alcohol, tobacco and recreational drugs is dependent upon the recall of the subject. One could speculate that NHL cases may tend to over-report the amount of substances used. The absence of any positive association for tobacco and alcohol, however, provides reassurance that over-reporting is not the sole factor behind the associations found.

The precise mechanism of the development of NHL among recreational drug users remains unknown. However, cocaine is associated with lymphocyte activation, and may be associated with chronic antigenic stimulation (Matsui et al, 1993). In the setting of on-going proliferation, and stimulation of the immune system, as well as by drug-induced aberrations of normal immunity, a random chromosomal error could occur. An error such as a translocation could result in the activation of certain oncogenes or deletion of tumour-suppressor genes, which might allow a selective growth advantage to a particular clone of B lymphocytes leading eventually to B-NHL (Gaidano and Dalla-Favera, 1992). Such a mechanism has been proposed for the AIDS-related lymphomas, in which chronic B-cell stimulation and proliferation are induced by HIV itself, as well as by the inflammatory cytokines (IL-6; IL-10) that are released as a consequence of HIV infection (Nakajima et al, 1989; Levine, 1992; Masood et al, 1994). In this setting of polyclonal B cell proliferation, an accidental translocation between chromosome 8 and chromosome 14 has been associated with dysregulation of c-*myc*, and the eventual development of small non-cleaved lymphoma (Subar et al, 1988; Ballerini et al, 1992; Nakamura et al, 1993). Other specific genetic errors have also been described, leading to other pathological types of AIDS–NHL (Gaidano et al, 1994).

Although recreational drug use alone, and in particular, cocaine use, will not explain the overall increase in NHL in the US, and although we cannot rule out confounding as an explanation for our finding, it is possible that use of these substances may explain some of the increase in incidence. Of note, the recent, more rapid increase in NHL incidence, which occurred even in areas with few AIDS-related cases, coincides with the epidemic of recreational drug use in the US (Devesa and Fears, 1992; Edlin et al, 1994). Further study is indicated to determine the specific mechanisms whereby recreational drugs might contribute to the risk of NHL, and to determine other factors which might explain the significant increase in NHL in the United States.

### ACKNOWLEDGEMENT

This work was supported in part by the PHS, National Institute of Health, National Cancer Institute Grant No. R01 CA-50850

### REFERENCES

Armenian HK, Hoover DR, Rubb S, Metz S, Martinez-Masa O, Chmiel J, Kingsley L and Saah A (1996) Risk factors for non-Hodgkin's lymphomas in acquired immunodeficiency syndrome (AIDS). *Am J Epidemiol* **143**: 374–379

Ballerini P, Gaidano G, Gong JZ, Tazzi V, Saglio G, Knowles DM and Dalla-Favera R (1992) Molecular pathogenesis of HIV-associated lymphomas. *AIDS Res Hum Retroviruses* **8**: 731–735

Boring CC, Squires TS and Tong T (1991) Cancer statistics, 1991. *CA Cancer J Clin* **41**: 19–36

Breslow NE and Day NE (1980) Statistical methods in cancer research, Vol 1, pp. 249–279. IARC Scientific Publications: Lyon

Brown LM, Gibson R, Burmeister LF, Schuman LM, Everett GD and Blair A (1992) Alcohol consumption and risk of leukemia, non-Hodgkin's lymphoma, and multiple myeloma. *Leuk Res* **16**: 979–984

Devesa SS and Fears T (1990) Non-Hodgkin's lymphoma time trends: United States and international data. *Cancer Res* **52** (suppl): 5432s–5440s

Edlin BR, Irwin KL, Faruque S, McCoy CB, Word C, Serrano Y, Inciardi JA, Bowser BP, Shilling RF and Holmberg SD (1994) Intersecting epidemics – crack cocaine use and HIV infection among inner city young adults. *N Engl J Med* **331**: 1422–1427.

Franceschi S, Serraino D, Carbone A, Talamini R and La Vecchia C (1989) Dietary factors and non-Hodgkin's lymphoma: a case–control study in the northeastern part of Italy. *Nutr Cancer* **12**: 333–341

Gaidano G and Dalla-Favera R (1992) Biologic aspects of human immunodeficiency virus related lymphoma. *Curr Opin Oncol* **4**: 900–906

Gaidano G, Lo Coco F, Ye BH, Shibata D, Levine AM, Knowles DM and Dalla-Favara R (1994) Rearrangements of the bcl-6 gene in Acquired Immunodeficiency Syndrome-associated non-Hodgkin's lymphoma: associate with diffuse large cell subtype. *Blood* **84**: 397–402

Hays HM, Tarone RE and Cantor KP (1991) Case–control study of canine malignant lymphoma: Positive association with dog owner's use of 2,4-dichlorophyeoxyacetic acid herbicides. *J Natl Cancer Inst* **63**: 1226–1231

Institute of Medicine (1993) *Veterans and Agent Orange: Health Effects of Herbicides used in Vietnam*. National Academy of Sciences: Washington DC

Isomaki HA, Hakulinen T and Joutsenlahti U (1979) Lymphoma and rheumatoid arthritis (letter). *Lancet* **1**: 392

Kassam SS, Thomas TL, Moutsopoulos HM, Hoover R, Kimberly RP, Budman DR, Costa J, Decker JL and Chused TM (1978) Increased risk of lymphoma in Sicca syndrome. *Ann Intern Med* **89**: 888–892

Kinlen LJ (1985) Incidence of cancer in rheumatoid arthritis and other disorders after immunosuppressive treatment. *Am J Med* **78**: 44–49

Kinlen LJ (1992) Immunosuppressive therapy and acquired immunological disorders. *Cancer Res* **52**(suppl.): 5474s–5476s

Levine AM (1992) Acquired immunodeficiency syndrome-related lymphoma (review). *Blood* **80**: 8–20

Massod R, Lunardi-Iskandar Y, Moudgil T, Zhang Y, Law RE, Huang C, Puri RK, Levine AM and Gill PS (1994) IL-10 inhibits HIV-1 replication and is induced by Tat. *Biochem Biophys Res Commun* **202**: 374–383

Matsui K, Friedman H and Klein TW (1993) Molecular mechanisms associated with cocaine induced modulation of human T lymphocyte proliferation. *Adv Exp Med Biol* **335**: 127–134

Mueller NE, Mohar A and Evans A (1992) Viruses other than HIV and non-Hodgkin's lymphoma. *Cancer Res* **52**(suppl.): 5479s–5481s

© Cancer Research Campaign 1997

Nakajima K, Martinez-Maza O, Hirano T, Breen EC, Nishanian PG, Salazar-Gonzales JF, Fahey JL and Kishimoto T (1989) Induction of IL-6 production by human immunodeficiency virus. *J Immunol* **142**: 531–536

Nakamura H, Said JW, Miller CW and Koeffler HP (1993) Mutation and protein expression of p53 in acquired immunodeficiency syndrome-related lymphomas. *Blood* **82**: 731–735

Persson B, Fredriksson M, Olsen K, Boeryd B and Axelson O (1993) Some occupational exposures and risk factors for malignant lymphomas. *Cancer* **72**: 1773–1778

Ries LA, Hankey BF and Edwards BK (1990) Cancer Statistics Review, 1973–1987. *DHHS Pub. No. (NIH)* 90-2789. Government Printing Office: Washington, DC

Rosenberg SA, Berard CW, Brown BW, Burke J, Dorfman RF, Glatstein E, Hoppe RT, Simon R, Henry K, Lennert K, Lukes RJ, O'Conor G, Rappaport H, Hartsock R, Kruger G, Nanba K, Robb-Smith AH, Sacks M, Banfi A, Bloomfield C, Bonadonna G, DeLellis R, DeVita VT Jr, Frizzera G, Hu MS, Kaplan HS, Rilke F, Rosai J, Rudders RA, Warnke RA and Ziegler JL (1982) National Cancer Institute sponsored study of classifications of non-Hodgkin's lymphomas. Summary and description of a working formulation for clinical usage. *Cancer* **49**: 2112–2135

Subar M, Neri A, Inghirami G, Knowles DM and Dalla-Favera R (1988) Frequent c-myc oncogene activation and infrequent presence of Epstein–Barr virus genome in AIDS-associated lymphoma. *Blood* **72**: 667–671

© Cancer Research Campaign 1997